# Transdermal Electrical Neuromodulation for Anxiety and Sleep Problems in High-Functioning Autism Spectrum Disorder: Feasibility and Preliminary Findings

**DOI:** 10.3390/jpm11121307

**Published:** 2021-12-06

**Authors:** Stephen T. Foldes, Amanda R. Jensen, Austin Jacobson, Sarah Vassall, Emily Foldes, Ann Guthery, Danni Brown, Todd Levine, William James Tyler, Richard E. Frye

**Affiliations:** 1 Division of Research, Barrow Neurologic Institute at Phoenix Children’s Hospital, Phoenix, AZ 85016, USA; stephen.foldes@dignityhealth.org (S.T.F.); ajacobson2@phoenixchildrens.com (A.J.); dbrown4@phoenixchildrens.com (D.B.); 2 Division of Neurology, Barrow Neurologic Institute, Phoenix, AZ 85013, USA; 3 School of Biological and Health Sciences, Arizona State University, Tempe, AZ 85287, USA; wtyler@asu.edu; 4 Department of Child Health, University of Arizona College of Medicine Phoenix, Phoenix, AZ 85004, USA; 5 Section on Neurodevelopmental Disorders, Division of Neurology, Barrow Neurologic Institute at Phoenix Children’s Hospital, Phoenix, AZ 85016, USA; ajensen1@phoenixchildrens.com; 6 Division of Psychology, Vanderbilt University, Nashville, TN 37240, USA; sarah.g.vassall@Vanderbilt.Edu; 7 Speech and Hearing Science, Arizona State University, Tempe, AZ 85287, USA; elfoldes@gmail.com; 8 Division of Psychiatry, Barrow Neurologic Institute at Phoenix Children’s Hospital, Phoenix, AZ 85016, USA; aguthery@phoenixchildrens.com (A.G.); tlevine2@phoenixchildrens.com (T.L.)

**Keywords:** α-amylase, autism spectrum disorder, anxiety, cortisol, heart rate variability, neuromodulation, sleep anxiety, transdermal electrical neuromodulation, neurostimulation

## Abstract

Background: Autism spectrum disorder (ASD) is associated with anxiety and sleep problems. We investigated transdermal electrical neuromodulation (TEN) of the cervical nerves in the neck as a safe, effective, comfortable and non-pharmacological therapy for decreasing anxiety and enhancing sleep quality in ASD. Methods: In this blinded, sham-controlled study, seven adolescents and young adults with high-functioning ASD underwent five consecutive treatment days, one day of the sham followed by four days of subthreshold TEN for 20 min. Anxiety-provoking cognitive tasks were performed after the sham/TEN. Measures of autonomic nervous system activity, including saliva α-amylase and cortisol, electrodermal activity, and heart rate variability, were collected from six participants. Results: Self-rated and caretaker-rated measures of anxiety were significantly improved with TEN treatment as compared to the sham, with effect sizes ranging from medium to large depending on the rating scale. Sleep scores from caretaker questionnaires also improved, but not significantly. Performance on two of the three anxiety-provoking cognitive tasks and heart rate variability significantly improved with TEN stimulation as compared to the sham. Four of the seven (57%) participants were responders, defined as a ≥ 30% improvement in self-reported anxiety. Salivary α-amylase decreased with more TEN sessions and decreased from the beginning to the end of the session on TEN days for responders. TEN was well-tolerated without significant adverse events. Conclusions: This study provides preliminary evidence that TEN is well-tolerated in individuals with ASD and can improve anxiety.

## 1. Introduction

Autism spectrum disorder (ASD) is a behaviorally defined neurodevelopmental disorder with lifelong consequences that affects children during critical times in their lives [1]. The Centers for Disease Control and Prevention (CDC) estimates that ASD affects about 2% of children (1 in 54) in the United States (US) [2]. The only standard treatment for core ASD symptoms is behavioral therapy. Although behavioral therapy can be effective if started early in life [3,4], only a minority of children obtain optimal outcomes [5,6] and most require lifelong supportive care [7]. The economic burden of intense and continuous educational, medical and social support is impressive [8], with the lifetime social costs to date in the US estimated to be more than $7 trillion [9]. In addition, the child’s disability creates a spillover effect that decreases the quality of life of the entire family [10,11,12]. 

### 1.1. Co-Occurring Conditions Can Interfere with Daily Function

ASD is associated with many co-occurring medical conditions, including intellectual disability [13], epilepsy [14], gastrointestinal disorders [15], sleep disorders [16], ADHD [16], anxiety [16], irritability, self-injurious behavior and depression [17]. While these conditions are not considered core symptoms of ASD, they can limit the functional ability of the individual, preventing them from gaining optimal benefit from therapies. Anxiety [18,19] and sleep problems [20,21] are difficult-to-treat lifelong conditions that commonly start in childhood and continue into adolescence and adulthood. These conditions commonly result in difficultly with the transition into independence, as well as significantly decreasing the ability to function optimally in everyday life.

Anxiety disorders are estimated to affect 40% of children and adolescents with ASD [19], which is consistently higher than the prevalence in neurotypical (NT) children [22,23]. Anxiety results in the avoidance of social situations, further worsening social isolation, which is commonly associated with ASD [24]. This is particularly problematic during adolescence and young adulthood, when high-functioning individuals with ASD are expected to interact with others independently. For individuals with ASD who are low-functioning with limited communication, anxiety commonly drives aberrant behaviors, such as aggression and self-injurious behavior. These behaviors are commonly refractory to treatment and can lead to institutionalization, making finding effective treatments extremely important. Anxiety also has detrimental physical effects on health beyond affecting daily activities. For example, studies on the NT population have shown that unmanaged anxiety can breakdown biological functions, such as the immune [25,26] and cardiovascular [27] systems. 

Sleep problems are more common in children and adolescents with ASD compared to the NT population, with a prevalence up to of 82% and persistent across their lifespan [28]. Sleep problems commonly start in early life and are potentially an early warning sign of ASD [29,30]. In young adulthood they reduce quality of life [31] and are associated with unemployment [20]. As discussed in our recent review, sleep problems in individuals with ASD are associated with worse ASD symptom severity, communication and social function, and increased irritability, stereotypy, hyperactive, anxiety, aggression and inattention [32]. Poor sleep in individuals with ASD results in a spillover effect which decreases quality of life for both the individual and the family [12]. Successful treatment of sleep problems improves a wide variety of ASD symptoms, including daytime behavior and function [32] as well as quality of life [12].

### 1.2. Treatment Approaches to Modulate Physiological Drivers of Symptoms

Standard treatments for core and associated ASD symptoms in addition to co-occurring conditions usually include psychopharmacological management, which tends to provide a suboptimal result in many cases. Indeed, pharmacological approaches borrowed from the treatment of the NT population do not translate well regarding both efficacy and AE profile. This leaves a large knowledge gap in the efficacious and safe treatment for these detrimental co-morbidities.

While there are many effective ways to manage anxiety in the NT population, these approaches are limited in effectiveness in ASD. Pharmacological approaches suffer from AEs, such as drowsiness, blunting general affect, impairing attention and dependence. In fact, a Cochrane review found that the standard treatment for anxiety in NT individuals, SSRIs, were not only not effective for individuals with ASD but may do more harm than good [33]. Relaxation-based treatments [34] and exercise [35] can be beneficial but require investments of time and training that often prohibit compliance and can be challenging for young adults, especially with ASD. Thus, alternative approaches to treat anxiety are needed for children and adolescents with ASD.

Sleep problems may be refractory to treatment in many individuals with ASD. Although standard behavioral therapy is the first line for treatment, it tends to be ineffective in children with ASD [36]. Melatonin has good clinical trial evidence for use in children with ASD for sleep initiation, but not for sleep maintenance [32,36]. Good evidence for other pharmaceutical treatments is lacking [32]. In fact, in individuals with ASD, especially adolescents and young adults, sleep initiation and maintenance are often refractory to standard treatments and can interfere with their ability to function.

Perhaps the greatest limitation of many ASD treatments is that most medical interventions focus on symptomatic relief rather than targeting underlying pathophysiological mechanisms. Thus, a safe, effective and well-tolerated treatment that addresses underlying pathophysiological abnormalities in ASD could accelerate the achievement of optimal outcomes for a greater proportion of individuals with ASD. New approaches to managing anxiety and sleep quality could greatly enhance the quality of life and activities of daily living for children with ASD as they transition into adulthood and age out of services. 

Multiple studies have documented autonomic nervous system (ANS) imbalances in individuals with ASD with relative sympathetic overactivation, leading to hyper-arousal, anxiety [37] and poor sleep [38]. ANS imbalances are linked to clinical symptoms associated with ASD. For example, heart rate variability (HRV) is associated with gastrointestinal symptoms [39], and an atypical pupillary light reflex is linked to sensory symptoms [40]. 

### 1.3. An Alternative Treatment Approach to Modulate the Physiological Drivers of Anxiety and Sleep Disruption

Based on evidence from NT individuals with anxiety [41], and plausible biological mechanisms of action, we hypothesized that transdermal electrical neuromodulation (TEN) of the cervical nerves is a safe, effective, comfortable and non-pharmacological therapy to modulate the central nervous system for decreasing anxiety and enhancing sleep quality, leading to an improved quality of life for people with ASD. Most importantly, TEN has the potential to improve many symptoms associated with ASD by correcting underlying dysregulated pathways which modulate anxiety, wakefulness and autonomic dysregulation. In contrast to pharmacological treatments which modulate general neurotransmitter pathways, potentially leading to unnecessary off-target, non-specific effects that can cause AEs, TEN modulates specific dysregulation pathways and provides targeted neuromodulation to alter endogenous neurotransmitter pathways.

**Figure 1:**
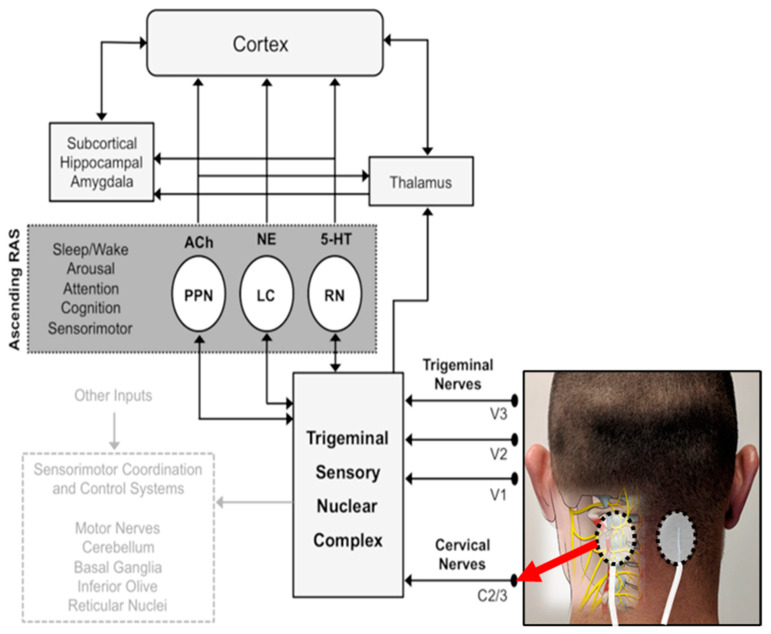
Mechanisms of electrical stimulation to improve anxiety and sleep. Cervical nerves are stimulated using a wearable transdermal neurostimulator placed on the back of the neck at the C2/C3 level, as shown. This modulates the ascending reticular activating system (RAS) via the trigeminal sensory nuclear complex. The ascending RAS includes the pedunculopontine nucleus (PPN), the locus coeruleus (LC) and raphe nuclei (RN), and modulates acetylcholine (Ach), norepinephrine (NE) and serotonin (5-HT) to higher-order brain structures to modulate attention and regulate awareness, arousal and sleep.

Using noninvasive electrical activation of peripheral, cervical or cranial nerves, TEN regulates the activity of several deep-brain nuclei within the ascending reticular activating system (RAS) [42,43] (Figure 1). RAS nuclei modulate the sympathetic nervous system by regulating norepinephrine (NE) from the locus coeruleus (LC), acetylcholine (Ach) from pedunculopontine nuclei (PPN) and serotonin (5-HT) from raphe nuclei (RN) [44,45]. These pathways have wide-ranging effects on cortical and subcortical brain regions, resulting in modulation of attention, awareness, arousal and sleep. 

TEN applied to the trigeminal or vagus nerves is therapeutic for ADHD [46], migraine headache [47], major depressive disorder [42], post-traumatic stress disorder [48] and generalized anxiety disorder [49], among others. Furthermore, it is consistently reported to be well-tolerated without any AEs. 

### 1.4. TEN May Be an Excellent Treatment for Anxiety and Sleep in ASD

Although TEN has not been used in ASD, the related technique of transcutaneous electrical acupoint stimulation was found to improve anxiety, general ASD and sensory symptoms in two controlled studies, with one study demonstrating improvement in plasma arginine-vasopressin levels [50,51]. Similarly, this technique has been shown to improve behaviors in valproic acid rodent models of ASD [52]. Interestingly, there is significant interest for the use of TEN in ASD [53]. This may lead to clinicians recommending these devices to children with ASD off-label, without any evidence for their effectiveness or best practices. It is therefore important to study TEN in order to provide evidence for effectiveness and define an optimal protocol. Given the unique nature of ASD, it is first important to assess the feasibility of TEN for this specific population, including sensitivity to electrodes and co-operation, as well as to confirm its safety. 

In our previous study [41], as compared to the sham, TEN significantly suppressed basal sympathetic tone, as measured by functional infrared thermography of facial temperatures, and lowered anxiety on the Profile of Mood States scale in healthy adults. With experimental stress, TEN produced a significant suppression of HRV, electrodermal activation (EDA) and salivary α-amylase levels compared to the sham. Thus, here we extended this treatment to individuals with ASD and anxiety.

## 2. Materials and Methods

This trial was registered on ClinicalTrials.gov as NCT03859336 and approved by the Phoenix Children’s Hospital (PCH) Institutional Review Board #19.265 (Phoenix, AZ). All parents of participants provided written informed consent. Those under 18 years of age also provided assent, and those older than 18 years of age provided consent. This study followed the CONSORT guidelines for pilot and feasibility trials [54]. The CONSORT checklist is available as Supplementary Table S1. This was planned as a one-year study, but was extended 6 months to facilitate recruitment because of COVID-19 restrictions put in place by Phoenix Children’s Hospital. 

### 2.1. Participants

Seven children and adolescents with high-functioning ASD and anxiety participated in this study (mean (SD) ages: 14.1 (4.5), 71% (5) males) (Table 1). They were recruited from the PCH Precision Medicine Autism Clinic, as well as social media advertisements.

Inclusion criteria: (1) previous diagnosis of ASD confirmed by the gold-standard instrument the Autism Diagnostic Interview–Revised (ADI-R) by a research reliable examiner; (2) age of 10–25 years old; (3) intellectual quotient > 80 on the Kaufman Brief Intelligence Test (KBIT-2); (4) Self- or parent-reported complaints of anxiety; (5) Screen for Child Anxiety Related Disorders–Parent (SCARED-P) form score ≥ 25; (6) able to follow directions in English; (7) ability to maintain all ongoing complementary, traditional and behavioral treatments during the study; and (8) no changes to any therapies for at least two months prior to time of participation.

Exclusion criteria: (1) implanted device; (2) history of electroconvulsive therapy; (3) history of significant face, head or neck injury, or surgery including metal plate or screw implants; (4) current or chronic neck pain; (5) pregnant or planning to become pregnant during the study period; (6) history of migraines or frequent headaches (more than once a week); (7) fainting (vasovagal syncope or neurocardiogenic syncope); (8) diagnosis of Raynaud’s disease; (9) temporomandibular joint disorder or other facial neuropathy; (10) poor vision or hearing that is uncorrectable; (11) epilepsy or seizures in the last 2 years; or (12) evidence of skin disease or skin abnormalities affecting the neck or upper back.

### 2.2. Study Design

**Figure 2:**
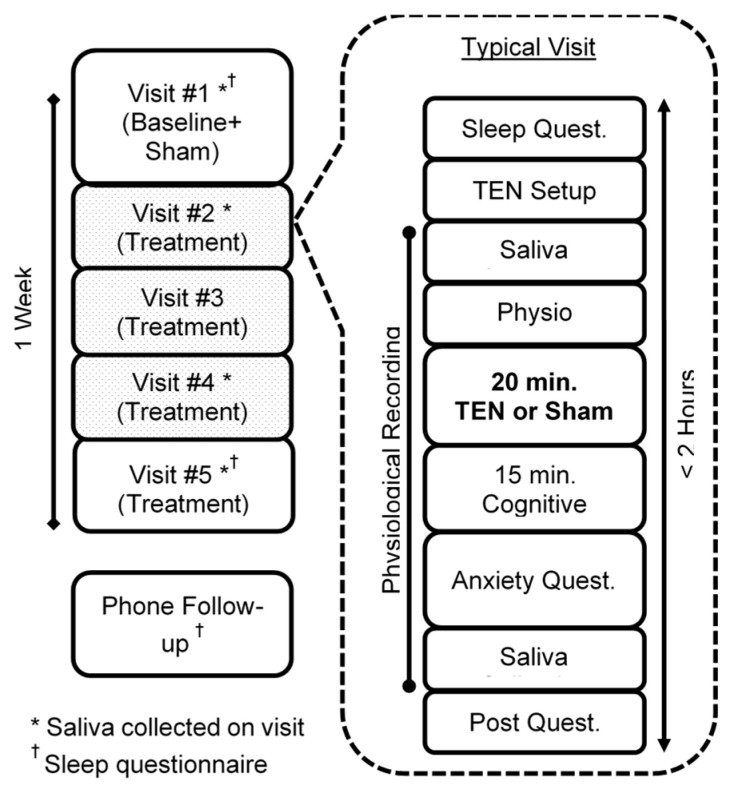
Study design.

This was a single-blinded sham-controlled study. Participants who qualified underwent 5 consecutive visits at the same time each day, receiving 20 min of the sham on day 1 and TEN on the next 4 consecutive days (See Figure 2). The participants and their parents were blinded to if or when they would receive the sham, only being told that they might receive the sham as part of the study. On day 1, screening procedures occurred in which the eligibility criteria, medical history and concomitant medications and therapies were reviewed. A urine hCG test was performed if the participant was female. Participants completed the KBIT-2. If their score was > 80 they qualified to continue on to the baseline session, as well as the 4 following sessions. 

Daily sessions began with the parent completing parent-rated questionnaires while the participant supplied a saliva sample and answered a pre-treatment AE questionnaire. TEN electrodes were taped to the back of the participant’s neck before a 5 min quiet physiological baseline period. Twenty minutes of the sham or TEN followed. After treatment, participants completed the ~15 min cognitive battery to stimulate anxiety, similar to that of a school test. The participant then answered the post-stimulation questionnaires to measure anxiety and any adverse events, as well as provided a second saliva sample. Saliva was collected on days 1, 2, 4 and 5. Sleep questionnaires were only performed on days 1 and 5. Parent-rated anxiety scales were completed every day of participation. During a follow-up phone call one week after study completion, anxiety and sleep symptoms were queried and a CSHQ was collected.

### 2.3. TEN Treatment

During treatment, bipolar electrical stimulation was delivered to the cervical plexus (C2-C4) on the back of the neck (see Figure 1). The stimulation electrodes were 1.25” (3.2 cm) round hydrogel PALS electrodes from Axelgaard Manufacturing Co., Ltd. (Fallbrook, CA, USA). Stimulation was delivered at a frequency of 300 Hz, pulse width of 350 ms and a duty cycle of 50% using a custom-made stimulation device. Stimulation amplitude was determined at every visit to ensure the current was just below the sensory threshold (see Table 2). To do this, the current amplitude was gradually increased until the participant’s sensation threshold was met, then the amplitude was set 0.5 mA lower, where the participant could not feel the stimulation. Stimulation was delivered at this amplitude for 20 min. For the day 1 sham, the same procedure was performed to determine the stimulation threshold, but no stimulation occurred for the 20 min. 

### 2.4. Outcome Measures

#### 2.4.1. Anxiety Measures

There are few adequate measures of anxiety for ASD, but the SCARED has excellent performance for the measurement of anxiety in ASD [55,56,57] with strong psychometrics, measurement invariance, test–retest reliability and external validity [58]. The PRAS-ASD is a recently developed tool for assessing anxiety in ASD [59]. In contrast to the parent-reported SCARED, there is no currently established thresholds for anxiety with the PRAS-ASD; therefore, the parent-reported SCARED was used in screening. 

#### 2.4.2. Sleep Measures

The CSHQ, the standard tool for assessing sleep problems in ASD [60], was given to parents at baseline, visit #5 and at one-week follow-up.

#### 2.4.3. Cognitive Battery

A ~15 min time-pressured cognitive battery was performed after stimulation/sham to replicate the anxiety in a test-taking situation, which is a common cause for anxiety in adolescents and young adults with ASD. The battery included three tests, given in the same order each day but with different question sequences depending on the day. The tests were explained to the participants before any stimulation on the first visit, and they were allowed to practice until they were comfortable. To account for cognitive differences, the task speed was faster for those with a high IQ (KBIT-2 score ≥ 100) and slower for those with a lower IQ (KBIT-2 score > 80–< 100). 

The first test was the Paced Auditory Serial Addition Test (PASAT) [61] where the participant listened to random single-digit numbers being spoken in increasingly rapid succession, added the two most recent numbers heard and verbally responded to the administrator with the sum. The test presented 91 numbers ranging from 0 to 9 with an interstimulus interval (ISI) that started at 4 s and decreased to 2 s for participants with a KBIT-2 score > 80–< 100, or started at 3 s and decreased to 1 s for participants with a KBIT-2 score ≥ 100. This task is well-known to increase anxiety [62] and has been used as an experimental inducer of psychological stress [63]. 

The next test was the symbol search task (SST), which required a participant to identify and count the number of target shapes found within a grid of shapes presented on a screen. The task consisted of 49 image grids made from 5 different shapes with similar features. The image grid started with a size of 3 × 3 and increased to 8 × 8 throughout the task. Image grids were presented in 6 blocks. Each block consisted of 8 image grids presented for *n* seconds each, where *n* was related to the size of the grid and the baseline IQ of the participant. For the easier version, a *n* × *n* shape array was shown for *n* seconds (e.g., at the end of the study an 8 × 8 grid was shown for 8 s), while for the harder version the array is shown for *n*-1 s (e.g., at the end of the study an 8 × 8 grid was shown for 7 s). Participants verbally reported their responses.

The final test was the n-back task (NBT), where a participant determined if a presented shape matched the shape presented 2 shapes earlier (2-back). The task consists of 50 sequential shapes chosen from 5 different shapes with similar features. Shapes were shown for 3 s with a 2 s ISI for participants with a lower IQ and 2 s with a 1 s ISI for participants with a higher IQ. This test is widely used to assess working memory, which is commonly impaired in ASD [64] 

Tasks were created using PsychoPy [65] and presented on a screen or speaker in front of the participant. A study coordinator sat behind the participant and scored their performance using prepared scoring sheets with the correct answers already indicated on them. 

#### 2.4.4. Autonomic Assessments 

Several outcome measures were evaluated to investigate the effect of TEN on the ANS. Salivary was collected by a study coordinator at the beginning and end of day 1 (sham) as well as days 2, 4 and 5. Samples were blindly analyzed by Salimetrics Laboratory (Carlsbad, CA, USA) for α-amylase and cortisol levels. Saliva was collected by passive drool. Participants were instructed to not brush their teeth within 45 min, eat within one hour, consume caffeine or alcohol within 12 h or have dental work performed within 24 h of their scheduled appointment. Participants were also rinsed their mouths before saliva collection. Samples were immediately stored at −20 °C, where they stayed until shipment to the laboratory for analysis.

Electrical activity across the heart was collected by bio-medical engineers using a Polar H10 device with a chest strap (Polar USA, Bethpage, NY, USA). This system has been evaluated in children [66]. The system calculated HRV from the R-R intervals using a proprietary algorithm. Data were streamed to our custom-coded data acquisition system and collated with other data sources. The changes in the average HRV were calculated during TEN/sham and during each test compared to the last 4 min of the baseline period. HRV is known to be associated with anxiety [67], and was quantified as the standard deviation of the normal-to-normal heartbeat interval.

EDA data were also gathered by bio-medical engineers; they were measured at the base or tip of the index and middle fingers using the Shimmer-3 GSR+ Unit (Dublin, Ireland). The phasic component of the EDA was computed using LedaLab with adaptive smoothing and continuous decomposition [68]. After processing with LedaLab, the maximum EDA values were extracted around each cognitive battery question (−2 to +2 s of question onset). The median EDA value across each test type was then computed as the overall EDA response to a test. During the cognitive battery, it was noted that participants’ anxiety levels were different for different tests, and during some challenging tests they would quit their effort early. To account for this, we considered only the first half of the questions (i.e., up to question 45, 24 and 25 for PASAT, SST and NBT, respectively). We also assessed the change in EDA across the day for the test that elicited the strongest EDA response for each individual on their initial visit. This represented the most stressful test for the individual. Several studies have used EDA to quantify the sympathetic response resulting from anxiety in ASD [37,38].

#### 2.4.5. Measurement of Adverse Events

In addition to daily parent interviews, adverse events were monitored by using pre- and post-treatment questionnaires. These questions included potential symptoms, including headache, neck pain, tingling, itchiness, sleepiness, difficulty paying attention, unusual feelings/attitudes/emotions, nausea, tense muscles, dizziness, anxious/worried/nervous, forgetful, heart beating loudly, sweating, abnormal sleep or seizures. These symptoms were reviewed both at the beginning and end of every session. 

### 2.5. Statistical Analysis

All variables were examined for normality using probability–probability plots, and link functions were adjusted to account for variables which deviated from normality. Ordinal questionnaire data were best represented by a Poisson loglinear link function, while other outcome variables were best represented by a normal distribution. 

In general, a generalized linear model (GLM) was used for analysis as implemented in SPSS PASW release 18.0.0 (IBM, Armonk, NY, USA), using the appropriate link function as mentioned above. The GLM is calculated using maximal likelihood estimation with 100 maximum interactions and 5 maximal step-having, absolute convergence criteria of 10^−5^ for a change in parameter estimates and a singularity tolerance of 10^−11^. The GLM calculates parameter significance by determining the change in the variance of the model as a parameter is added, essentially determining if adding the parameter to the model significantly increases the variance accounted for by the model. This is represented by the chi-square statistic. The effect size, φ, is calculated from the chi-square statistic as $\sqrt{x^{2}/n}$, where *n* is the number of observations. Given that χ^2^ is a one-sided asymmetric distribution, only the lower bound of the effect size is calculated, which is provided in parathesis after the effect size. This was calculated using the chisq_to_phi function provided by the ‘effectsize’ library in R version 4.1.1 (The R Foundation for Statistical Computing, Vienna, Austria). For φ 0.1 is consider a small effect, 0.3 is considered a medium effect and 0.5 is considered a large effect.

Analysis using the GLM followed two approaches. First, a continuous variable that represented a linear increase in effect over treatment days was used to represent a change in effect with additional treatment days in order to identify a dose effect. Second, a dichotomous treatment effect (no treatment vs. treatment) represented whether treatment of all days combined was different than the sham. For salivary measures that were collected before and after the testing session, a dichotomous effect (before vs. after) factor was added to examine the change over the experimental session as well as the interaction of this factor with the treatment effect variable. For the responder analysis, the interaction of the dichotomous responder factor (responder vs. non-responder) with the treatment effect was examined. For a further illustration of individual measures as treatment progresses, please refer to Supplemental Figure S1 and Supplemental Figure S2. 

## 3. Results

Thirteen individuals were screened for participation from January 2020 to July 2021. Six individuals did not pass the screening. Three did not meet the anxiety threshold, one’s IQ was too low, one had changed anxiety medication with <6 weeks prior to the study and one could not be contacted when starting the study. Of the participants that started the study, none discontinued the study. A CONSORT flow diagram is presented as Supplementary Figure S1.

### 3.1. Adverse Effects

There were no significant AEs reported during the trial. Participants were specifically asked about the comfort of electrode placement and stimulation. No participant reported discomfort from the study procedures, including the electrodes. After completing the trial, one participant who had a history of precocious puberty and periodic oligomenorrhea experienced oligomenorrhea. The participant had stopped her medication to suppress menses 3 months prior to the study. It is not known whether TEN triggered the recurrent oligomenorrhea. 

### 3.2. Anxiety 

**Figure 3:**
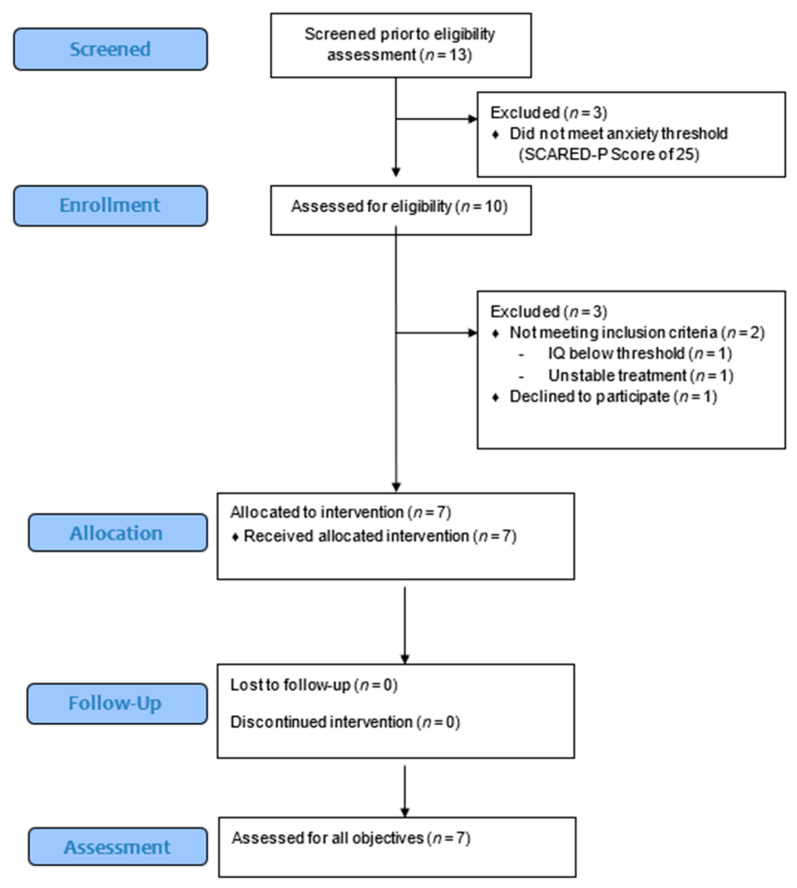
CONSORT flow diagram illustrating the allocation of each subject. Thirteen subjects were screened and 7 underwent treatment and assessment for all objectives.

**Figure 4:**
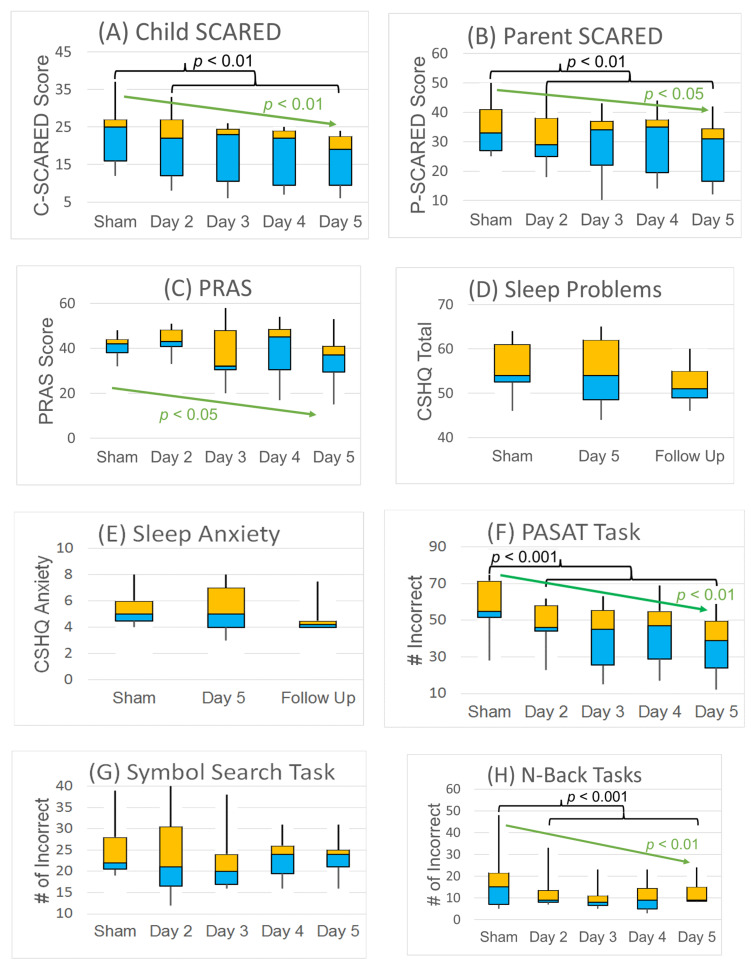
Effects of TEN treatment on (**A**–**C**) anxiety, (**D**,**E**) sleep and (**F**–**H**) cognitive performance (*n* = 7). Median, quartiles and min and max are shown. Black brackets represent significant differences between the sham day and TEN days (days 2–5). Green arrows represent significant trends in outcome with more treatment days.

In general, the three anxiety questionnaires demonstrated progressive improvement in overall anxiety over 4 days of TEN treatment as compared to the sham. Over the group, the C-SCARED improved from a median of 25 to 19 (i.e., 24%), while the P-SCARED improved from a median of 33 to 31 (i.e., 7%). As seen in Supplemental Figure S1A, all participants demonstrated an improvement in the C-SCARED score at the last TEN day, with all ending with scores below the clinical cutoff (i.e., <25). The C-SCARED demonstrated a dose effect with a progressive reduction in score with increasing days of TEN (χ^2^(1) = 9.86, *p* < 0.01; φ = 0.53 (0.25)), and was reduced for all treatment days combined (χ^2^(1) = 7.39, *p* < 0.01; φ = 0.45 (0.18)) (Figure 3 and Figure 4A). 

As seen in Supplemental Figure S1B, six of the seven (86%) participants demonstrated an improvement on the P-SCARED, with three (43%) ending with scores below the clinical cutoff (i.e., <25). The P-SCARED also demonstrated a dose effect with a progressive reduction in score with increasing days of TEN (χ^2^(1) = 7.69, *p* < 0.01; φ = 0.47 (0.19)), and was reduced for all treatment days combined (χ^2^(1) = 5.70, *p* = 0.02; φ = 0.40 (0.12)) (Figure 4B). 

As seen in Supplemental Figure S1C, the PRAS decreased in five of the seven participants (71%) and, as a group, demonstrated a significantly greater effect with increasing days of TEN treatment (χ^2^(1) = 4.09, *p* < 0.05; φ = 0.34 (0.04)), but was not significantly different for overall effect of all days combined (Figure 4C).

### 3.3. Sleep

As seen in Supplemental Figure S1D–E, five of the seven (71%) participants improved in total CSHQ score, and five of the seven (71%) participants improved in CSHQ anxiety score. From the CSHQ, sleep anxiety and total score decreased the week following TEN although these changes did not reach statistical significance (Figure 4D,E). The CSHQ decreased from a median of 54 to 51 (5%) in overall symptoms, and the sleep anxiety subscale decreased from 6 to 4 (33%) one week following TEN treatment. These findings are consistent with unsolicited feedback from multiple parents reporting improved sleep following the study.

### 3.4. Anxiety-Provoking Task Performance

As seen in Supplementary Figure S1F, all of the participants demonstrated improvement on the PASAT, with the median number of incorrect responses declining from 55 to 39 for the PASAT, a 29% improvement with a very large effect size. PASAT performance significantly improved with an increasing number of TEN sessions (χ^2^(1) = 39.57, *p* < 0.001; φ = 1.06 (0.79)), and was better on the days with TEN as compared to the sham (χ^2^(1) = 29.91, *p* < 0.001; φ = 0.92 (0.65)) (Figure 4F). As can be seen in Supplementary Figure S1G, six of the seven (86%) improved performance on the SST, with the median number of incorrect answers declining from 22 to 24. There was no significant change in the number of incorrect answers on the SST (Figure 4G). As can be seen in Supplementary Figure S1H, only three of the seven (43%) showed improvement and one of the seven (14%) demonstrated no change on the NBT. However, given the large improvements in one particular participant, the median number of incorrect answers declined from 15 to 9, a 40% improvement with a large effect size. NBT performance significantly improved with more TEN sessions (dose effect) (χ^2^(1) = 9.43, *p* < 0.01; φ = 0.52 (0.24)), and was better on the days with TEN as compared to the sham (χ^2^(1) = 16.75, *p* < 0.001; φ = 0.69 (0.41)) (Figure 4H). 

### 3.5. Salivary Biomarkers

**Figure 5:**
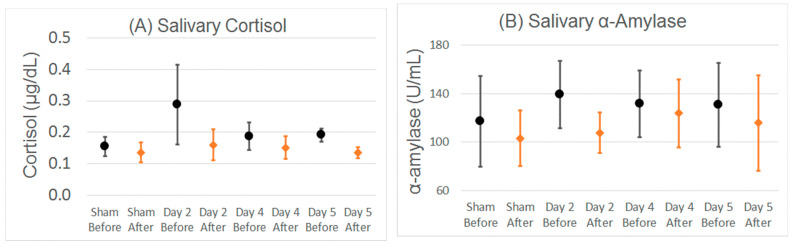
Salivary (**A**) cortisol and (**B**) α-amylase changes before and after TEN/sham and anxiety-provoking tasks. Mean and standard error bars are shown. There were no statistically significant differences between the sham and TEN days, or before as compared to after TEN/sham and anxiety-provoking tasks for the whole group.

As can be seen in Supplementary Figure S2A-B, across the group salivary α-amylase and cortisol levels varied considerably, and were not significantly different between the sham and TEN days, did not significantly change with more TEN and did not significantly change over each experimental day (Figure 5).

### 3.6. Heart Rate Variability

**Figure 6:**
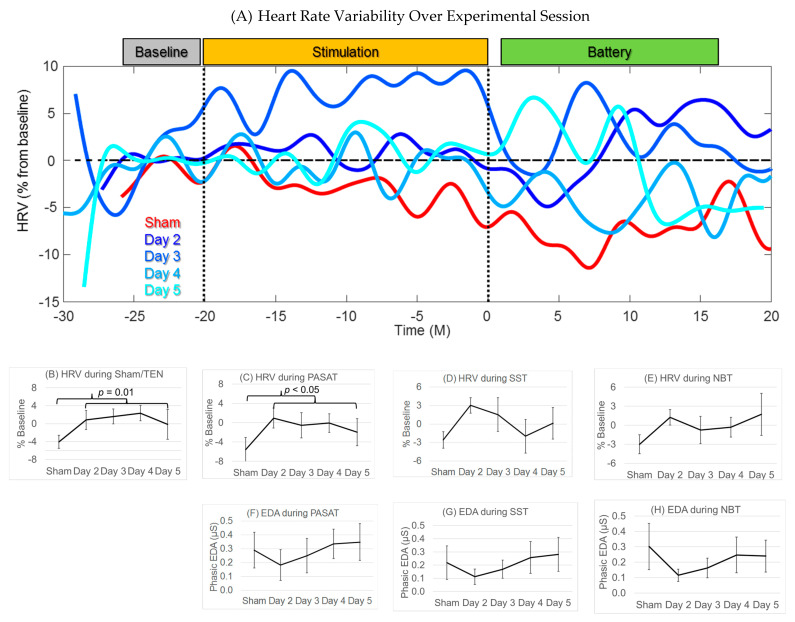
The effect of TEN on HRV and EDA. Mean and standard error bars are shown. The increase in HRV is shown for (**A**) an example individual, (**B**) across the group for the time period of the sham/TEN and (**C**–**E**) and for the anxiety-provoking tasks. HRV was significantly higher (improved) during TEN sessions as compared to the sham during the (**B**) stimulation and (**C**) the PASAT (shown as black brackets). EDA during (**F**–**H**) anxiety-provoking tasks decreased after the sham, but did not reach significance at the group level. No EDA was computed during the sham/TEN.

Figure 6A demonstrates the HRV changes over experimental sessions for one example participant. From this figure it is obvious that the change in HRV is different for the sham vs. the experimental sessions. During sham stimulation (red line) HRV decreases (becomes more pathologic), and then continues to decrease during the anxiety-inducing tasks, demonstrating the stressful effects of the cognitive battery. On the first day of TEN treatment (dark blue; day two), the HRV increases with TEN (improved autonomic balance). Individual responses for HRV variability are shown in Supplementary Figure S2C–F. HRV was significantly higher on TEN treatment days as compared to the sham during the stimulation period (χ^2^(1) = 6.37, *p* = 0.01, φ = 0.46 (0.16); Figure 6B) and the PASAT (χ^2^(1) = 4.74, *p* < 0.05, φ = 0.40 (0.09); Figure 6C). Although this trend was also seen during SST (Figure 6D) and the NBT (Figure 6E), the difference was not statistically significant. The effect of TEN on HRV did not appear to progressively change across days of stimulation.

### 3.7. Electrodermal Activity

We have compared the phasic EDA response during the anxiety-provoking tasks between the sham and TEN treatment days. Individual responses for EDA are shown in Supplementary Figure S2G,H. Overall, there was a consistent pattern to the effect of TEN on EDA. On the first day there was a decrease in phasic EDA response (Figure 6F–H). However, the EDA appeared to return to baseline levels after the first stimulation day. There was no significant difference for treatment vs. the sham, and no progressive effect over increasing days of treatment. 

### 3.8. Effect of Intelligence

The effect of IQ on baseline measures was evaluated. A higher IQ was associated with higher anxiety scores on the baseline C-SCARED (χ^2^(1) = 9.76, *p* < 0.01, φ = 1.18 (0.56)) and P-SCARED (χ^2^(1) = 4.16, *p* < 0.05, φ = 0.77 (0.11)), but not PRAS. A higher IQ was associated with better baseline performance on the PASAT (χ^2^(1) = 7.06, *p* < 0.01, φ = 1.00 (0.38)), but was not related to performance on the NBT or SST. A higher IQ was related to a higher overall salivary α-amylase (χ^2^(1) = 7.29, *p* < 0.01, φ = 1.10 (0.43)) at baseline, but not related to salivary a-amylase change or cortisol. IQ was not associated with changes in the C-SCARED, CSHQ or task performance across sessions, but it was related to improvements in anxiety rated with the P-SCARED (χ^2^(1) = 6.38, *p* = 0.01, φ = 0.43 (0.15)) and PRAS (χ^2^(1) = 8.40, *p* < 0.01, φ = 0.49 (0.21)) such that higher IQ was associated with more moderate ratings of improvement in anxiety on these parent-rated scales.

### 3.9. Responder Analysis

Results for participants who responded well to the TEN treatments compared to the sham (responders) were compared to the results from non-responders. Responders were defined as participants with an average of ≥ 30% improvement in C-SCARED with treatment compared to the sham. Four participants (57%) were categorized as responders and three (43%) as non-responders. There were no significant differences in the anxiety or sleep questionnaires at baseline between responders and non-responders, but responders performed worse on the SST (χ^2^(1) = 5.04, *p* < 0.05, φ = 0.85 (0.22)) and the PASAT (χ^2^(1) = 13.05, *p* < 0.001, φ = 1.37 (0.74)) during the baseline (sham) day. There were no differences in salivary cortisol or a-amylase between responders and non-responders.

Unsurprisingly, responders demonstrated a greater improvement (i.e., decrease) in the C-SCARED (χ^2^(2) = 11.25, *p* < 0.01, φ = 0.57 (0.29)) than non-responders. This difference increased with more treatments (χ^2^(1) = 9.99, *p* < 0.01, φ = 0.53 (0.26)). For those considered responders, the median C-SCARED dropped from 23 to 15 (35%), whereas the median C-SCARED dropped from 12 to 8 (4%) for the non-responders. For responders, the change in the improvement in the C-SCARED ranged from a minimum drop from 12 to 8, a 33% drop, to a maximum drop from 18 to 6, a 67% drop. For non-responders, the change in the C-SCARED ranged from a minimum drop from 25 to 24, a 4% drop, to a maximum drop from 14 to 11, a 21% drop. The median P-SCARED dropped from 32.5 to 24 (27%) for responders, but only dropped from 33 to 31 (6%) for non-responders. For responders, the change in the improvement in the P-SCARED ranged from a minimum increase from 29 to 34, a 17% increase, to a maximum drop from 36 to 14, a 61% drop. For non-responders, the change in the P-SCARED ranged from a minimum drop from 33 to 31, a 6% drop, to a maximum drop from 46 to 35, a 24% drop. However, there were no significant differences between groups in the PRAS-ASD or sleep questionnaires. The median PRAS-ASD dropped from 40 to 33 (18%) for responders, but only dropped from 43 to 38 (12%) for non-responders. For responders, the change in the improvement in the PRAS-ASD ranged from a minimum increase from 42 to 53, a 27% increase, to a maximum drop from 38 to 15, a 61% drop. For non-responders, the change in the PRAS-ASD ranged from a minimum increase from 32 to 38, a 19% increase, to a maximum drop from 43 to 30, a 30% drop. The median total CSHQ sleep score dropped from 56 to 53 (16%) for responders, and dropped from 54 to 47 (13%) for non-responders. For responders, the change in the improvement in the total CSHQ sleep score ranged from a minimum increase from 52 to 56, an 8% increase, to a maximum drop from 63 to 51, a 19% drop. For non-responders, the change in the total CSHQ sleep score ranged from a minimum unchanged score of 46, a 0% change, to a maximum drop from 54 to 47, a 13% drop. The median total CSHQ sleep anxiety score dropped from 5.5 to 4 (17%) for responders, and dropped from 5 to 4 (20%) for non-responders. For responders, the change in the improvement in the total CSHQ sleep anxiety score ranged from a minimum of unchanged at 4, a 0% increase, to a maximum drop from 6 to 4, a 33% drop. For non-responders, the change in the total CSHQ sleep anxiety score ranged from a minimum unchanged score of 4, a 0% change, to a maximum drop from 5 to 4, a 20% drop.

**Figure 7:**
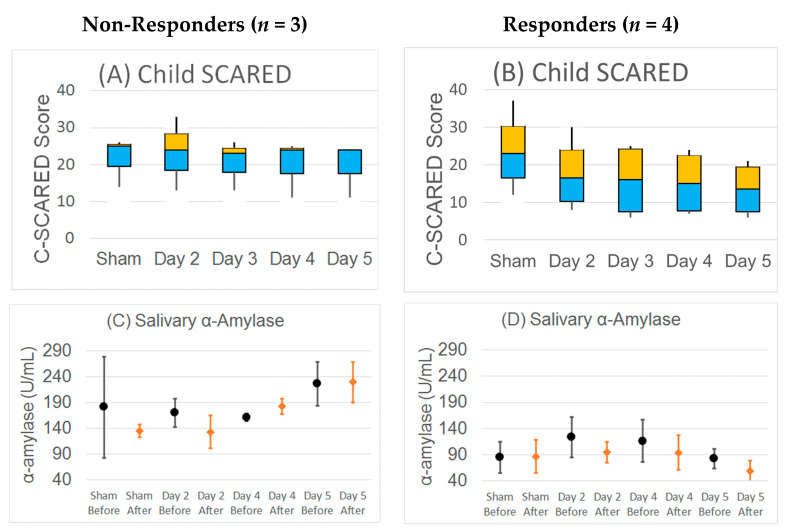
Responder analysis. Responders were defined as participants with an average of ≥30% improvement in the child-rated SCARED with treatment compared to the sham. In non-responders, the (**A**) child-rated SCARED did not change over sessions and (**C**) salivary α-amylase increased with more TEN sessions. In contrast, in responders the (**B**) child-rated SCARED markedly decreased and (**D**) α-amylase not only decreased with more TEN stimulation across multiple sessions, but also decreased from the beginning of the session to the end of the session on days the participants received TEN. For the child-rated SCARED median, quartiles and min and max are shown. For salivary α-amylase mean and standard error bars are shown.

Average daily salivary α-amylase became progressively higher across days in two of the non-responders, while it stayed stable and then decreased for the four responders (χ^2^(2) = 27.02, *p* < 0.001, φ = 0.67 (0.46)). In the responders salivary α-amylase within the day decreased after TEN treatment, as compared to before TEN treatment, whereas in non-responders it did not show a consistent change and even increased during day four of the study (χ^2^(2) = 26.73, *p* < 0.001, φ = 0.67 (0.46)) (Figure 7). There was no difference in change in cortisol for responders and non-responders.

## 4. Discussion

TEN is a well-tolerated method for subthreshold neurostimulation that is FDA-approved for brain-based disorders such as ADHD [46] and migraines [47], and also appears to have some effectiveness in major depressive disorder [42], post-traumatic stress disorder [48] and generalized anxiety disorder [49]. This is the first study to investigate the tolerability and effectiveness of TEN therapy in ASD, a disorder with many co-morbidities including anxiety and sleep disruption. In this study, adolescents with high-functioning ASD and anxiety underwent four daily TEN treatments following an initial sham run-in treatment day. Anxiety was monitored using standardized participant and caregiver questionnaires, sleep was monitored with caregiver questionnaires and multiple measures of ANS activity were collected including salivary cortisol and α-amylase, HRV and EDA during anxiety-provoking tasks and HRV during TEN. 

The treatment was well-tolerated with no reported AEs, consistent with studies of transcutaneous electrical acupoint stimulation in which children with ASD find the treatment tolerable, without AEs [50,51]. This study demonstrates the feasibility of TEN for individuals with ASD. This is reassuring as many suffer from hypersensitivity to tactile stimuli, making them unable to tolerate electrodes placed on their skin, as well as have symptoms of ADHD, which makes them unable to sit still for prolonged periods of time. These data suggest that TEN is a promising treatment for individuals with ASD that can be tolerated for a typical stimulation period. 

### 4.1. TEN Improved Rating of Anxiety in ASD

Across the group, anxiety scores were improved on treatment days as compared to the sham when considering both the participant- and caregiver-rated SCARED. Similarly, more days of stimulation had greater improvement in anxiety in all three anxiety questionnaires. The C-SCARED improved by 24% (from 25 to 19) across days, while the P-SCARED improved by 7% (from 33 to 31). Given the cutoff for anxiety is 25 for the SCARED, these results demonstrate that TEN had a clinically significant improvement in anxiety on the group level. In fact, all of the seven participants had a C-SCARED below the threshold for symptoms of an anxiety disorder after TEN treatment. This impact was even stronger for those considered responders, where the C-SCARED dropped 35% from 23 to 15 and the P-SCARED dropped 27% from 32.5 to 24. The PRAS drop in total score from 42 at baseline to 37 on day five. The effect sizes varied from a large effect of 0.53 for the C-SCARED to a medium effect of 0.34 for the PRAS. 

Previous studies suggest that a 55% decrease in the P-SCARED and a 50% decrease in the C-SCARED optimally predicted treatment response in a 6 month study of the SSRI sertraline and/or cognitive behavior therapy in NT youths [69]. Although this study did not demonstrate this level of treatment response, the 4-day treatment period was much shorter in duration. Thus, assessing a longer term treatment will be necessary to understand if the effect of TEN is cumulative. The results suggest TEN treatment could have a cumulative effect on improving anxiety in ASD. Although the questionnaire demonstrated a positive response to treatment, questionnaires are subjective, and studies have shown that they do not capture anxiety differences equivalently for all individuals with ASD [70]. More objective measurements of anxiety would improve future studies.

### 4.2. Possible Effect of TEN on Sleep in ASD

Though the improvements in sleep questionnaire scores did not reach significance, the findings were promising. We were especially encouraged by several participants’ unsolicited feedback, reporting improvement in sleep after TEN treatment. We did find that the CSHQ decreased three points (5%) in overall symptoms and two points (33%) in the sleep anxiety subscale one week following TEN treatment. These improvements in CSHQ scores are similar to those reported in other studies of sleep treatment in ASD. For example, three weeks of transcranial direct current stimulation to the left dorsal lateral prefrontal cortex was associated with a statistically significant 2.7-point drop in the total CSHQ score in the treatment group of a single-blinded, randomized, parallel clinical study of children with ASD [71], and a meta-analysis found that non-pharmacological interventions for insomnia in children with ASD was associated with a 4.71-point improvement in the CSHQ [72]. In contrast, one-month of controlled-release melatonin in children with ASD is associated with a 20-point improvement in the total CSHQ score and a 3.5-point improvement on the sleep anxiety scale in an open-label study [73].

Sleep problems are complicated to treat and may require prolonged treatment to measure improvements. These results are encouraging following only 5 days of treatment; a longer follow-up may be necessary to determine the full effect of treatment. An additional limitation of our sleep analysis is the use of the well-validated, but subjective, CSHQ. As a secondary reporter measure, it may not accurately capture the sleep quality of the participant. The addition of more objective measures of sleep, such as actigraphy, would provide a reliable index of sleep quality and quantity. Actigraphy utilizes established technology and algorithms to measure sleep onset and duration, as well as the number and duration of nighttime wakings. 

### 4.3. TEN Improved Performance on Anxiety-Provoking Tasks

To test cognitive performance, we utilized three anxiety-provoking tasks. These tasks involve executive function, which is not only commonly impaired in high-functioning adolescents with ASD but is also a key deficit that impedes performance in a wide variety of everyday tasks. It should be noted that this is the first time anxiety-provoking tasks have been used in a treatment trial of anxiety for individuals with ASD. Such assays should be considered as part of the assessment for anxiety treatments in other studies, given the importance of executive function skills in performance in academia, social interactions and general skills of daily living.

TEN was associated with a substantial improvement in performance for two of the three anxiety-provoking tasks. The median number of incorrect responses declined from 55 to 39 for the PASAT, a 29% improvement with a very large effect size, and declined from 15 to 9 for the NBT, a 40% improvement with a large effect size. The PASAT has been used in clinical trials for multiple sclerosis, traumatic brain injury and gerontology where the score improvements with treatment were smaller than seen in this study [61,74]. 

For the SST, the only anxiety-provoking task without a significant improvement in performance, the median number of incorrect answers increased from 22 to 24, an actual worsening of performance over the week. Analysis of the individual scores indicated that several of the participants performed worse on day four and five as compared to day three, which parallels the feedback provided from participants who reported they became uninterested with the task after performing it several times. This could indicate a complete lack of anxiety to successfully complete the task. Future studies might integrate other techniques, such as eye tracking, to help monitor performance and engagement.

### 4.4. Cortisol Response to Social Paradigms as a Bio-Marker for Stress in ASD

Cortisol is considered a measure of stress, commonly used to measure levels of general stress in many studies. However, in our study we did not find that it was a reliable biomarker of anxiety. This may be related to the ASD population, as other studies have also not found it to be a reliable measure of stress with those with ASD. Studies examining the responsiveness of cortisol in ASD also demonstrate an overall under-reactivity involving social threats [75,76,77], with this hyporeactive cortisol response to a social stressor greater in those with ASD and anxiety than compared to those with ASD and controls [78]. Some studies have shown greater cortisol reactivity in those with ASD as compared to controls to a nonsocial stressor [79], but not others [80,81]. Thus, a blunted cortisol response is not completed unexpected for the ASD group, especially those with anxiety. In addition, cortisol levels might be less likely to change with TEN as it is regulated through the hypothalamic–pituitary–adrenal axis which has multiple levels of potential dysregulation not directly linked to immediate sympathetic and parasympathetic neuromodulation. 

### 4.5. α-Amylase Response to Social Paradigms as a Bio-Marker for Stress in ASD

As α-amylase levels are directly related to sympathetic nervous system activation, it was expected that TEN would modulate α-amylase as a marker of ANS sympathetic activity. Although changes in α-amylase were not significant across the entire group of participants, it did seem to be different for those that responded to the treatment as compared to those that did not respond. For the responders to TEN, the α-amylase salivary concentrations showed a steady decrease across days of treatment, with the α-amylase salivary concentration decreasing following TEN treatment with each day of treatment. This decrease in α-amylase suggests a decrease in sympathetic activity with TEN treatment for the responder group. In contrast, those that did not respond to TEN had α-amylase levels that increased progressively on each successive day of stimulation, and the measured change within each day did not correlate with treatment. While we interpret this as an indication of decreased anxiety due to TEN treatment, this biomarker may be representing something more basic about modulation of the ANS. Indeed, the increase in α-amylase in the non-responders may have indicated that the TEN treatment parameters used in this study are not tuned to positively modulating the ANS in these specific participants, and that other TEN parameters may be needed for these participants to respond. Further research is needed to validate α-amylase as a potential biomarker of response to TEN and determine which levels of the psychological and/or physiological response it may represent.

### 4.6. TEN Improved Heart Rate Variability in ASD

We found that HRV significantly increased (i.e., improved) with TEN treatment, although this change was not progressive over days. This suggests that TEN positively impacted sympathetic regulation and that HRV may be a promising outcome measure for future studies. HRV has been used as a measure of ANS activity in both adults [82,83,84,85] and children [39,86,87,88] with ASD. Lower HRV has been associated with symptoms of gastrointestinal problems, such as constipation in children with ASD [39]. HRV has also been used as an outcome measures in clinical trials of yoga and repetitive transcranial magnetic stimulation in children with ASD [88,89,90]. Thus, HRV appears to be a promising biomarker of the ANS in children with ASD. 

### 4.7. Dynamic Changes in Autonomic Nervous System Measures with TEN 

Some ANS measures positively peaked only on the first day of TEN and then reverted to baseline. These include EDA during all tasks and HRV during the SST. Although this could be interpreted as the effect of TEN wearing off after the first day, such a notion is counter to the continuing positive effect of TEN on anxiety measurements and task performance. An alternative explanation is that this might demonstrate homeostatic mechanisms adapting to the TEN effect on the ANS and compensating for the effect of TEN on the ANS. This demonstrates some of the difficulties in using ANS measures for proxies of anxiety or other cognitive states, as they are regulated by multiple higher-level and lower-level influences. Furthermore, this return to baseline in ANS measures across days may also indicate that TEN is resetting the brainstem circuitry, and thereby facilitating a calmer and more consistent reaction to anxiety-provoking stimuli. 

### 4.8. Further Refinement of TEN Protocol

This study suggests that TEN may be useful for short-term improvement of anxiety, but long-term use of this device for individuals with ASD has not been studied. Extended long-term use is important, particularly with respect to the stimulation protocol. For responders, positive changes in C-SCARED (Figure 7B) and task performance (Figure 4F,H) as well as changes in ANS measures ( Figure 5 and Figure 6) were strongest after the first TEN treatment. From our studies it is not clear if consecutive-day treatment is necessary, nor whether it is optimal for stimulation to occur every week or every month to maintain and enhance the effect. Electrical stimulation regiments for other disorders vary considerably. Furthermore, only a single set of stimulation parameters were evaluated. Though the stimulation parameters used in this study are based on studies of healthy adults [41], personalized or group-wise optimization could help increase the portion of responders. Determining the optimal stimulation protocol for long-term use will help maximize benefits and minimize burdens. Once that is established, long-term studies can be designed to determine efficacy. Furthermore, improved biomarkers of the response to treatment will help refine future studies and maximize the effect size of treatment. 

### 4.9. Limitations

This study used subjective measures of anxiety, but subjective measures are standard for clinical care and clinical trials. This weakness was mitigated by obtaining both participant and observer (parent) measures. Individuals with ASD by definition have trouble communicating, so it is important to obtain a measure of their experience. Physiological measures relating to ANS activity were also used as a proxy for anxiety, but such measures are only preliminary in their ability to measure psychological states. Future studies should concentrate on validating reliable outcome measure to provide the most accurate representation of the effectiveness of TEN. 

This study was limited to high-functioning individuals with ASD, as communication is necessary to assess anxiety. Interestingly, IQ was related to perceived improvement in anxiety on the parent-rated scales, suggesting that the ability of the individual with ASD to communicate complex emotions may have influenced the rating of anxiety as well as its changes during the study.

A procedural limitation was that the sham day was always on the first day of participation. This was meant as a run-in to check for a placebo effect, but future studies should include a longer treatment period, with sham days dispersed throughout. Participants, as well as the researchers who are scoring the participant, should be blinded to the status of the stimulation.

## 5. Conclusions

Applying tuned, high-frequency stimulation to the cervical nerves appears to be a promising non-pharmacological, biologically informed, non-invasive and safe neuromodulation for managing anxiety in individuals with ASD. Such a treatment could potentially be particularly impactful for young people transitioning into adulthood, where anxiety and sleep problems can be exacerbated by time-based testing and the increased need to interact socially in order to function independently. With recent interest in TEN treatment for ASD, the expanding use of these devices in many disorders, the increased availability of these devices, and the well-established history of its safety, it would not be surprising if these devices were being recommended ‘off-label’ without any ASD-specific studies to guide their use. This study provides some insight into the safety and effectiveness of TEN for ASD. While there is preliminary evidence suggesting effectiveness in some of the individuals in our study, further studies are needed to better understand its benefits and develop an optimal protocol for its use.

## 6. Patents

Existing patents for the stimulation technology are held by Tyler (WO2013192582A1, US8903494B2 and US9002458B2).

## Figures and Tables

**Table 1:** Participant characteristics. Baseline Kaufman Brief Intelligence Test-2 (KBIT-2), child-rated Screen for Child Anxiety Related Disorders (C-SCARED), parent-rated Screen for Child Anxiety Related Disorders (P-SCARED), Parent-Rated Anxiety Scale (PRAS) and Total and Sleep Anxiety Score of the Children’s Sleep Health Questionnaire (CSHQ).

						Baseline		
Study ID	Age (Years)	Gender	KBIT-2 Score	SCARED—Child	SCARED—Parent	PRAS	Total CSHQ	Sleep Anxiety
TEN-01	12	Female	103	14	25	43	46	4
TEN-03	13	Male	83	12	25	38	63	6
TEN-05	10	Male	116	25	46	48	64	8
TEN-06	21	Male	109	26	33	32	54	5
TEN-07	20	Male	104	28	29	42	59	6
TEN-08	10	Male	96	18	36	38	52	5
TEN-09	13	Female	109	37	50	45	53	4

**Table 2:** TEN parameters.

Study ID	Frequency (Hz)	Duty Cycle	Pulse Width (ms)	Daily Threshold (Milliamps)				
				Day 1	Day 2	Day 3	Day 4	Day 5
TENS-01	300	50%	350	2.5	2.0	3.5	3.0	3.0
TENS-03	300	50%	350	4.5	4.0	5.5	6.5	5.5
TENS-05	300	50%	350	2.5	2.0	2.0	2.5	2.5
TENS-06	300	50%	350	2.5	3.5	3.5	2.5	4.5
TENS-07	300	50%	350	0.5	3.0	1.5	1.0	1.5
TENS-08	300	50%	350	3.5	13.5	13.5	12.5	8.5
TENS-09	300	50%	350	2.0	3.0	1.0	1.0	1.0

## Data Availability

Data and protocol are availability upon request.

## Supplementary Materials

The following are available online at https://www.mdpi.com/article/10.3390/jpm11121307/s1, Supplemental Table S1: CONSORT checklist; Supplemental Figure S1: Individual anxiety and sleep measures. Supplemental Figure S2: Individual autonomic measures.
