# Supplementary material for: Transdermal Electrical Neuromodulation for Anxiety and Sleep Problems in High-Functioning Autism Spectrum Disorder: Feasibility and Preliminary Findings
Source: J Pers Med. 2021 Dec 6;11(12):1307. doi: 10.3390/jpm11121307 (PMC8704341; doi:10.3390/jpm11121307)
Supplement: Supplementary file 1 [file jpm-11-01307-s001.zip › Supplemental Figure S1.pdf]

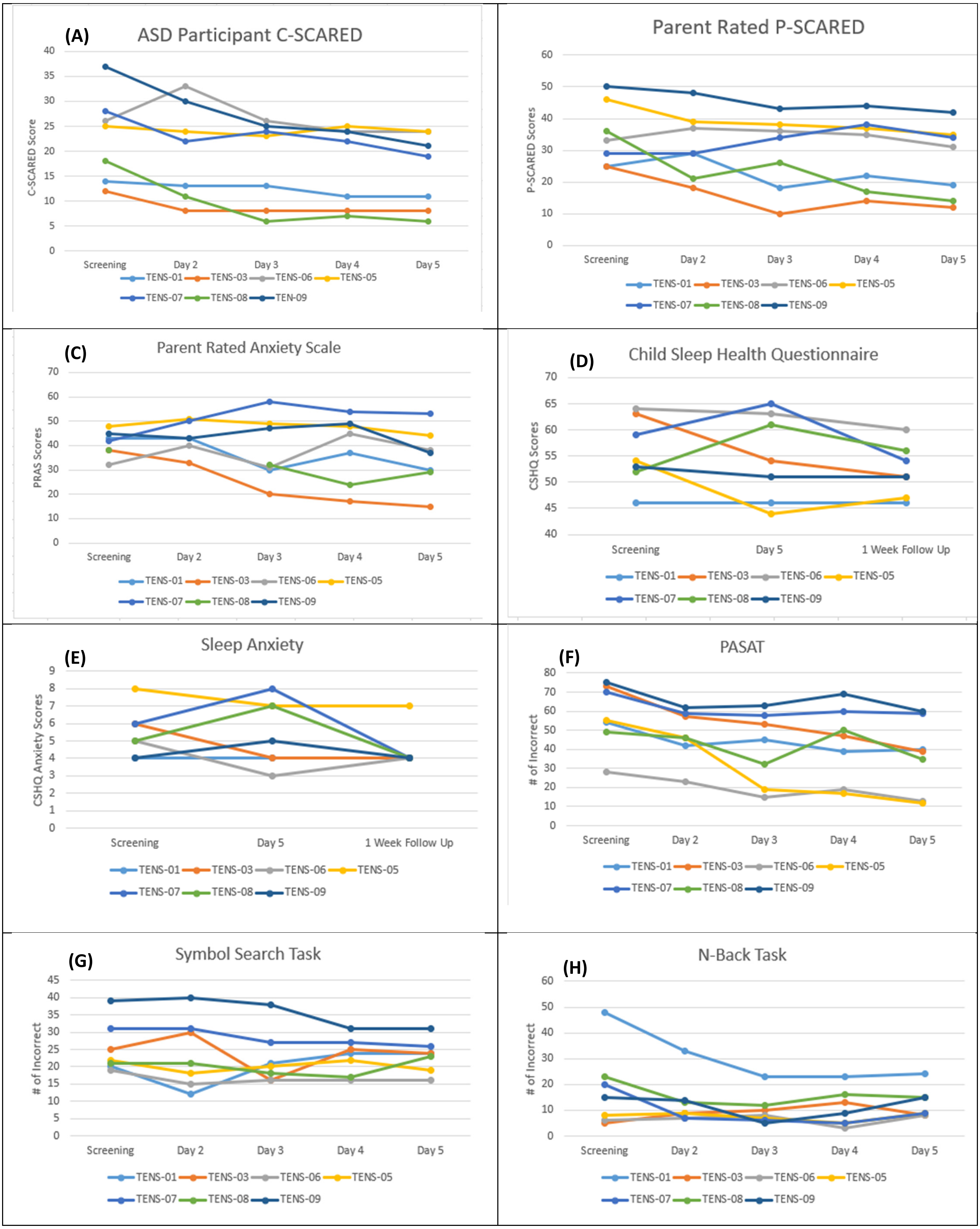

Figure S1: Individual distribution of scores of (A) C-SCARED, (B) P-SCARED, (C) PRAS, (D) CSHQ, (E) Sleep Anxiety, as well as number of incorrect scores on the (F) PASAT, (G) Symbol Search and (H) N-Back tasks.

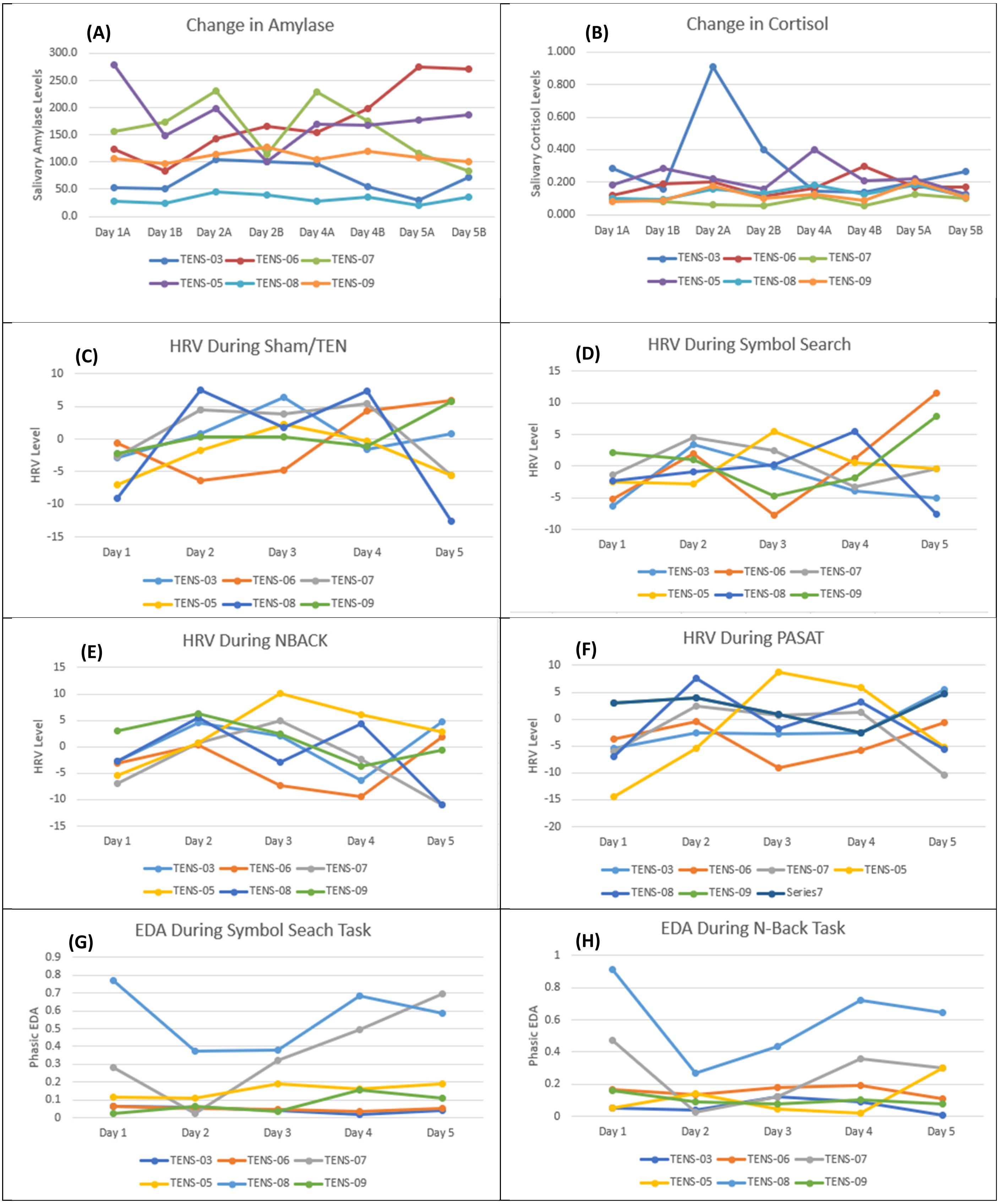
(A)
Change in Amylase
Salivary Amylase Levels
300.0
250.0
200.0
150.0
100.0
50.0
0.0
Day 1A
Day 1B
Day 2A
Day 2B
Day 4A
Day 4B
Day 5A
Day 5B
TENS-03
TENS-06
TENS-07
TENS-05
TENS-08
TENS-09
(B)
Change in Cortisol
Salivary Cortisol Levels
1.000
0.800
0.600
0.400
0.200
0.000
Day 1A
Day 1B
Day 2A
Day 2B
Day 4A
Day 4B
Day 5A
Day 5B
TENS-03
TENS-06
TENS-07
TENS-05
TENS-08
TENS-09
(C)
HRV During Sham/TEN
HRV Level
10
5
0
-5
-10
-15
Day 1
Day 2
Day 3
Day 4
Day 5
TENS-03
TENS-06
TENS-07
TENS-05
TENS-08
TENS-09
(D)
HRV During Symbol Search
HRV Level
15
10
5
0
-5
-10
Day 1
Day 2
Day 3
Day 4
Day 5
TENS-03
TENS-06
TENS-07
TENS-05
TENS-08
TENS-09
(E)
HRV During NBACK
HRV Level
15
10
5
0
-5
-10
-15
Day 1
Day 2
Day 3
Day 4
Day 5
TENS-03
TENS-06
TENS-07
TENS-05
TENS-08
TENS-09
(F)
HRV During PASAT
HRV Level
10
5
0
-5
-10
-15
-20
Day 1
Day 2
Day 3
Day 4
Day 5
TENS-03
TENS-06
TENS-07
TENS-05
TENS-08
TENS-09
Series7
(G)
EDA During Symbol Seach Task
Phasic EDA
0.9
0.8
0.7
0.6
0.5
0.4
0.3
0.2
0.1
0
Day 1
Day 2
Day 3
Day 4
Day 5
TENS-03
TENS-06
TENS-07
TENS-05
TENS-08
TENS-09
(H)
EDA During N-Back Task
Phasic EDA
1
0.8
0.6
0.4
0.2
0
Day 1
Day 2
Day 3
Day 4
Day 5
TENS-03
TENS-06
TENS-07
TENS-05
TENS-08
TENS-09

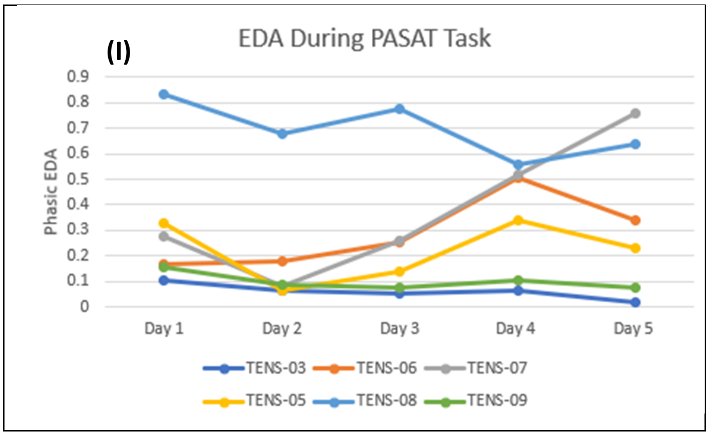

Figure S2: Individual progression of all autonomic measures including (A) Amylase, (B) Cortisol, (C) HRV during Sham/TEN Treatment, (D) HRV during Symbol Search task, (E) HRV during N-Back task, (F) HRV during PASAT task, (G) EDA during Symbol Search task, (H) EDA during N-Back task and (I) EDA during PASAT task.
